# Plasma Oxylipin Profile Discriminates Ethnicities in Subjects with Non-Alcoholic Steatohepatitis: An Exploratory Analysis

**DOI:** 10.3390/metabo12020192

**Published:** 2022-02-19

**Authors:** Tagreed A. Mazi, Kamil Borkowski, Oliver Fiehn, Christopher L. Bowlus, Souvik Sarkar, Karen Matsukuma, Mohamed R. Ali, Dorothy A. Kieffer, Yu-Jui Y. Wan, Kimber L. Stanhope, Peter J. Havel, John W. Newman, Valentina Medici

**Affiliations:** 1 Department of Nutrition, University of California Davis, Davis, CA 95616, USA; tmazi@ucdavis.edu (T.A.M.); pjhavel@ucdavis.edu (P.J.H.); john.newman2@usda.gov (J.W.N.); 2 Division of Clinical Nutrition, Department of Community Health Sciences, College of Applied Medical Sciences, King Saud University, Riyadh 11433, Saudi Arabia; 3 West Coast Metabolomic Center, Genome Center, University of California Davis, Davis, CA 95616, USA; kborkowski@ucdavis.edu (K.B.); ofiehn@ucdavis.edu (O.F.); 4 Division of Gastroenterology and Hepatology, Department of Internal Medicine, University of California Davis, Sacramento, CA 95817, USA; clbowlus@ucdavis.edu (C.L.B.); ssarkar@ucdavis.edu (S.S.); dorothy.a.kieffer@gmail.com (D.A.K.); 5 Department of Pathology and Laboratory Medicine, University of California Davis, Sacramento, CA 95817, USA; kmatsukuma@ucdavis.edu (K.M.); yjywan@ucdavis.edu (Y.-J.Y.W.); 6 Department of Surgery, University of California Davis, Sacramento, CA 95817, USA; mrali@ucdavis.edu; 7 Department of Molecular Biosciences, School of Veterinary Medicine, University of California Davis, Davis, CA 95616, USA; klstanhope@ucdavis.edu; 8 United States Department of Agriculture-Agriculture Research Service-Western Human Nutrition Research Center, Davis, CA 95616, USA

**Keywords:** arachidonic acid, Caucasian, cyclooxygenases, endocannabinoids, ethnicity, Hispanic, lipoxygenases, non-alcoholic steatohepatitis, oxylipins, polyunsaturated fatty acids, soluble epoxide hydrolases

## Abstract

Non-alcoholic fatty liver disease (NAFLD) is a common liver pathology that includes steatosis, or non-alcoholic fatty liver (NAFL), and non-alcoholic steatohepatitis (NASH). Without a clear pathophysiological mechanism, it affects Hispanics disproportionately compared to other ethnicities. Polyunsaturated fatty acids (PUFAs) and inflammatory lipid mediators including oxylipin (OXL) and endocannabinoid (eCB) are altered in NAFLD and thought to contribute to its pathogenesis. However, the existence of ethnicity-related differences is not clear. We employed targeted lipidomic profiling for plasma PUFAs, non-esterified OXLs and eCBs in White Hispanics (HIS, *n* = 10) and Caucasians (CAU, *n* = 8) with biopsy-confirmed NAFL, compared with healthy control subjects (HC; *n* = 14 HIS; *n* = 8 CAU). NAFLD was associated with diminished long chain PUFA in HIS, independent of histological severity. Differences in plasma OXLs and eCBs characterized ethnicities in NASH, with lower arachidonic acid derived OXLs observed in HIS. The secondary analysis comparing ethnicities within NASH (*n* = 12 HIS; *n* = 17 CAU), confirms these ethnicity-related differences and suggests lower lipoxygenase(s) and higher soluble epoxide hydrolase(s) activities in HIS compared to CAU. While causes are not clear, these lipidomic differences might be with implications for NAFLD severity and are worth further investigation. We provide preliminary data indicating ethnicity-specific lipidomic signature characterizes NASH which requires further validation.

## 1. Introduction

Non-alcoholic fatty liver disease (NAFLD) is a chronic liver condition affecting one in four adults worldwide and this rate increases with coexisting components of metabolic syndrome [1]. Its histological presentation includes hepatocellular steatosis, or non-alcoholic fatty liver (NAFL) with a range of necroinflammation with or without fibrosis. When hepatocellular damage and ballooning are present, this is clinically defined as non-alcoholic steatohepatitis (NASH) [2]. The pathogenesis of NAFLD is not fully elucidated. Whereas its onset involves an interplay between genetics and environmental factors with coexisting comorbidities, the progression to NASH appears to be provoked by multiple or parallel hits including oxidative stress and inflammation [3,4]. Oxidative stress modulates insulin signaling, lipid metabolism, inflammation, and fibrogenesis, and many oxidative stress biomarkers have been associated with NAFLD severity [5,6]. In the U.S., the risk and severity of NAFLD vary among ethnic/racial groups, with Hispanics (HIS) being affected disproportionately and presenting more frequently with advanced inflammation and fibrosis compared to other ethnicities [7,8,9]. The metabolic drivers underlying this disparity are not clear.

Polyunsaturated fatty acid (PUFAs) are bioactive lipids and precursors to inflammatory lipid mediators including oxylipins (OXLs) and endocannabinoids (eCBs). OXLs are produced from PUFAs by mono- and dioxygenases, including lipoxygenases (e.g., 5-LOX, 12-LOXs, and 15-LOXs); cyclooxygenases (i.e., COX-1 and -2), and a variety of cytochrome P450s (CYPs) [10]. PUFAs can also undergo non-enzymatic oxygenation mediated by free radicals and the rate of this production is increased under oxidative stress [11]. In general, OXLs from n-3 PUFAs have anti-inflammatory or less pro-inflammatory effect compared to those derived from n-6 PUFAs [12,13]. The fatty acid ethanolamides (i.e., N-acylethanolamides), one class of eCB, are synthesized by complex interactions of lipases and fatty acid amide hydrolase from PUFAs and membrane associated precursors [14]. Collectively, these lipids work through receptor-mediated mechanisms and likely contribute to NAFLD by modulating processes including lipogenesis, inflammation, and mitochondrial β-oxidation [10,15].

Previous lipidomic analyses showed that NAFLD is associated with dysregulated PUFAs metabolism [16,17,18,19,20]. Alterations in circulating OXL and eCB profiles are reported in NAFLD and other liver pathologies. In fact, numerous lipid mediators have been shown to predict NAFL or NASH [18,21,22,23,24,25,26]. However, metabolomic profiling in NAFLD with regards to ethnicity is limited. Our prior semi-quantitative lipidomic profiling study indicated ethnicity-specific differences in plasma PUFA profiles in subjects with NAFL, with higher abundance of linoleic acid (LA) and α-linolenic acid (ALA) seen in Caucasians (CAU) compared to ethnicity-matched lean subjects [27]. In the same study, the progression to NASH was characterized by ethnicity-specific differences in hepatic lipidomic profiles with higher levels of saturated and unsaturated fatty acids seen in NASH-HIS. Ethnicity was not previously addressed in OXL and eCB profiling efforts. Examining such lipidomic differences among ethnicities may shed light on potential mechanisms modulating the disparity in NAFLD prevalence and severity.

The objective of this “proof-of-concept” study is to examine ethnicity-related changes in PUFAs and their downstream inflammatory mediators in a group of subjects with obesity and biopsy-confirmed NAFL and NASH. We employed targeted lipidomic analysis of plasma PUFAs, OXLs, and the N-acylethanolamides class of eCBs to compare HIS and CAU subjects with medically complicated obesity to ethnicity-matched lean healthy controls (HC). Profiles in subjects diagnosed with NASH were also compared to ethnicity and BMI-matched participants without NASH (0-NASH). In addition, we conducted a secondary analysis including prospectively collected subjects to compare OXL profile between ethnicities in NASH.

## 2. Results

### 2.1. Subject Characteristics

The clinical and histological features of NAFL subjects from the primary cohort are presented in Table 1. The mean age in NAFL and HC, respectively, was 47 ± 15 and 43 ± 14 in HIS; 50 ± 18 and 44 ± 12 in CAU (n.s). The mean BMI in HIS was 46 ± 6 in NAFL and 26 ± 2 in HC (*p*-value < 0.05); in CAU, the mean BMI was 42 ± 8 in NAFL and 25 ± 3 in HC (*p*-value < 0.05). Within NAFL group, the mean NAS score was 3 ± 3 and 3 ± 1 for HIS and CAU, respectively (n.s). No difference in clinical and histological parameters was found between ethnicities. In subjects with NASH compared to 0-NASH, the mean NAS score was 5 ± 2 and 4 ± 1 for HIS and CAU, respectively (n.s) (data not shown).

The secondary cohort included NASH subject with various degrees of necroinflammation and fibrosis (Table 2). When comparing NASH-HIS and NASH-CAU, no difference was found with BMI and other clinical and histological parameters. The mean NAS score was 5 ± 2 and 5 ± 1 for NASH-HIS and NASH-CAU, respectively (n.s).

### 2.2. Ethnicity-Related Alterations in Plasma PUFAs and Lipid Mediator Profiles Characterize NAFL

**Figure 1:**
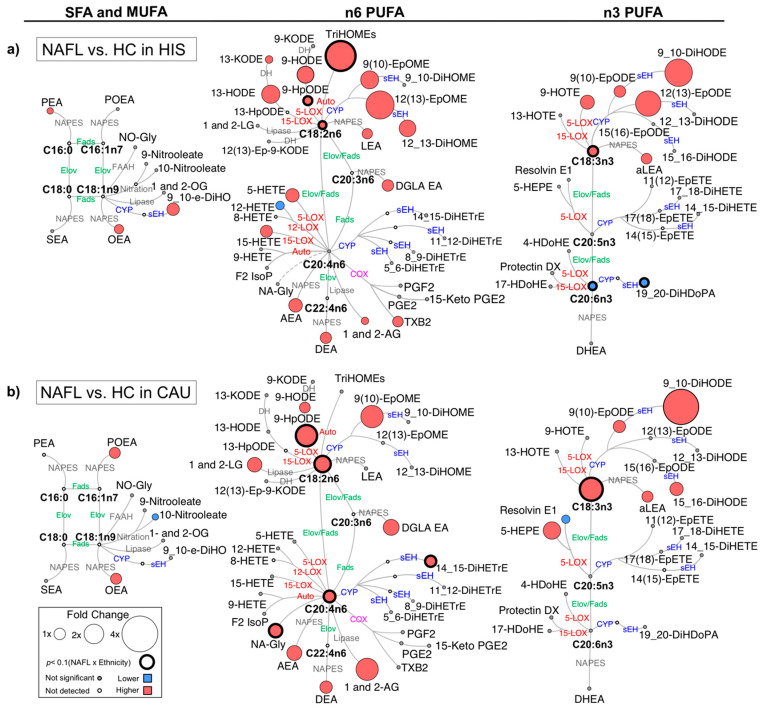
Differences in plasma polyunsaturated fatty acids (PUFAs) and lipid mediators between non-alcoholic fatty liver (NAFL) compared to healthy control (HC) in primary cohort. Metabolic network for (**a**) Hispanic (HIS); (**b**) Caucasian (CAU) illustrating saturated (SFAs), monounsaturated (MUFAs) and polyunsaturated fatty acids (PUFAs), including n-3 and n-6 PUFAs with pathways of oxylipins and endocannabinoids synthesis. Node size represents fold changes, calculated as (HC − NAFL)/HC. Node’s color represents the directionality of differences: higher in NAFL (red); lower in NAFL (blue); no change (grey). Shown are only lipids with differences between NAFL vs. HC (*t*-test raw *p* < 0.05) and/or with interaction (ethnicity × NAFL) (ANCOVA raw *p* < 0.05). Lipids with interaction (ethnicity × NAFL) (ANCOVA raw *p* < 0.05) are marked with a solid circle. Means and *p*-values are detailed in Table S1. Fatty acids are described by number of carbons and double bounds of the fatty acyl moiety (i.e., C18:2n6). NAFL (*n* = 10 HIS and 8 CAU); HC (*n* = 14 HIS and 8 CAU). ADH, alcohol dehydrogenase; AEA, arachidonoyl ethanolamine; AG, arachidonoyl glycerol; DEA, docosatetraenyl ethanolamide; DGLEA, dihomo-gamma-linolenoyl ethanolamide; DH, dehydrogenase; DHEA, docosahexaenoyl ethanolamide; DiHDoPA, dihydroxydocosapentaenoic acid; DiHETE, dihydroxyeicosatetraenoic acid; DiHETrE, dihydroxyeicosatrienoic acid; DiHO, dihydroxyoctadecanoic acid; DiHODE, dihydroxyoctadecadienoic acid; DiHOME, dihydroxyoctadecenoic acid; Elov, fatty acid elongase; Ep-KODE, epoxyoxooctadecenoic acid; EpETE, epoxyeicosatetraenoic acid; EpODE, epoxyoctadecadienoic acid; EpOME, epoxyoctadecenoic acid; F2-IsoP, F2 isoprostanes; FAAH, fatty acid amide hydrolase; Fads, fattyaciddesaturase; HDoHE, hydroxydocosahexaenoic acid; HEPE, hydroxyeicosapentaenoic acid; HETE, hydroxyeicosatetraenoic acid; HODE, hydroxyoctadecadienoic acid; HOTE, hydroxyoctadecatrienoic acid; HpODE, hydroperoxyoctadecadienoic acid; KETE, keto-eicosatetraenoic; KODE, keto-octadecadienoic acid; LEA, Linoleyl ethanolamine; LG, linoleoylglycerol; LOX, lipoxygenase; NA-Gly, arachidonylglycine; NAPES, N-acylphosphatidyl ethanolamine-specific; NO-Gly; OEA, oleoyl ethanolamine; OG, oleoylglycerol; PEA, palmitoyl ethanolamine; PGE, prostaglandin E; PGF, prostaglandin F; POEA, palmitoleoyl ethanolamide; SEA, stearoyl ethanolamide; sEH, soluble epoxide hydrolase; TriHOME, trihydroxyoctadecaenoic acid; TXB, thromboxane.

We examined differences in plasma fatty acids and lipid mediators between NAFL and HC (Figure 1 and Table S1). Compared to corresponding HC, 25 (38% of total detected) and 7 (11%) lipid levels were different in NAFL-HIS and NAFL-CAU, respectively (FDR-adjusted *p* < 0.2). Ethnicity-specific changes observed in NAFL, with interaction (ethnicity × NAFL), include 8 lipids (15%) and 2 enzymatic ratios (raw *p*-Interaction < 0.05) but did not survive FDR-correction (q = 0.2). To rule out any effect of histological severity on the differences observed between ethnicities, the analysis was repeated on a subset of histology-matched subjects (Table S2). As a result, 12 lipids (19%) and one enzymatic ratio were found altered (raw *p*-Interaction < 0.05), with 3 lipids, i.e., ALA, LA, and 9-hydroperoxyoctadecadienoic acid (-HpODE) surviving FDR correction.

When compared to HC, there were overlapping differences seen in NAFL for both ethnicities as well as ethnicity-specific differences (Figure S1). In both ethnicities, NAFL showed higher levels of several eCBs derived from PUFA and other fatty acids. The 18 carbon (C18) PUFAs, ALA and LA showed similar higher trend with higher levels of downstream fatty acid alcohols, hydroperoxide, ketones, epoxides, and vicinal diols. Specific to NAFL-HIS, there were differentially higher levels of LA-triols (i.e., trihydroxyoctadecaenoic acids (TriHOMEs) (raw *p*-Interaction < 0.05). On histology-matched analysis, higher TriHOMEs and LA-epoxide, 12(13)-epoxyoctadecenoic acid (-EpOME) levels were found significant (raw *p*-Interaction < 0.05). Of note, the n-6 to n-3 ratio was higher, however, with no ethnicity × NAFL interaction. In NAFL-CAU, there was differentially higher ALA and LA and its hydroperoxide, 9-HpODE (raw *p*-Interaction < 0.05). On histology-matched analysis, these lipids retained significance with LA, and 9-HpODE passing the FDR-threshold.

The 20 carbon (C20) and longer chain PUFAs (LC-PUFA) showed opposite trends with higher levels in NAFL-CAU and lower in NAFL-HIS. The ratio of docosahexaenoic acid (DHA)/eicosapentanoic acid (EPA) + ALA was found lower in both ethnicities (FDR-adjusted *p*-value). In HIS, alcohols, ketones, thromboxane derived from arachidonic acid (AA) were higher, however with no interaction (ethnicity × NAFL). DHA and its vicinal diol, 19,20-dihydroxydocosapentaenoic acid (-DiHDoPA), levels were differentially lower (raw *p*-Interaction < 0.05) with tendency shown for lower EPA. On histology-matched analysis, lower levels of these lipids were found significant (raw *p*-Interaction < 0.05). Specific to CAU, there was differentially higher AA and its vicinal diol, 14,15-dihydroxyeicosatrienoic acid (-DiHETrE) that remained significant after histology adjustment (raw *p* < 0.05). While these findings show common alterations seen in NAFL for both ethnicities, they also highlight ethnicity-specific changes. This includes a divergence in LC-PUFA profile, mainly with lower EPA and DHA seen in HIS. Although these differences did not pass FDR adjustment, histology-matched analysis yielded consistent and stronger differences, suggesting ethnicity-specific differences characterized NAFL, independently of liver histology severity. It also suggests that fibrosis may weaken the differences between ethnicities.

### 2.3. Ethnicity-Related Differences in Plasma PUFAs and Lipid Mediators’ Independent of Obesity

Ethnicity-specific differences in plasma lipidome within lean HC were examined (Figure S2 and Table S1). Among the differences observed, HIS had higher LA (raw *p* < 0.05), ALA, 9-HpODE, and TriHOMEs levels (FDR-adjusted *p*-value), and lower AA-derived prostaglandin, PGE2 (FDR-adjusted *p*-value) levels. These findings indicate alterations in plasma PUFAs and lipid mediator profiles in HIS independent of obesity.

### 2.4. The Progression to NASH Is Characterized by Ethnicity-Related Alterations in Plasma PUFAs and Lipid Mediator Profiles

**Figure 2:**
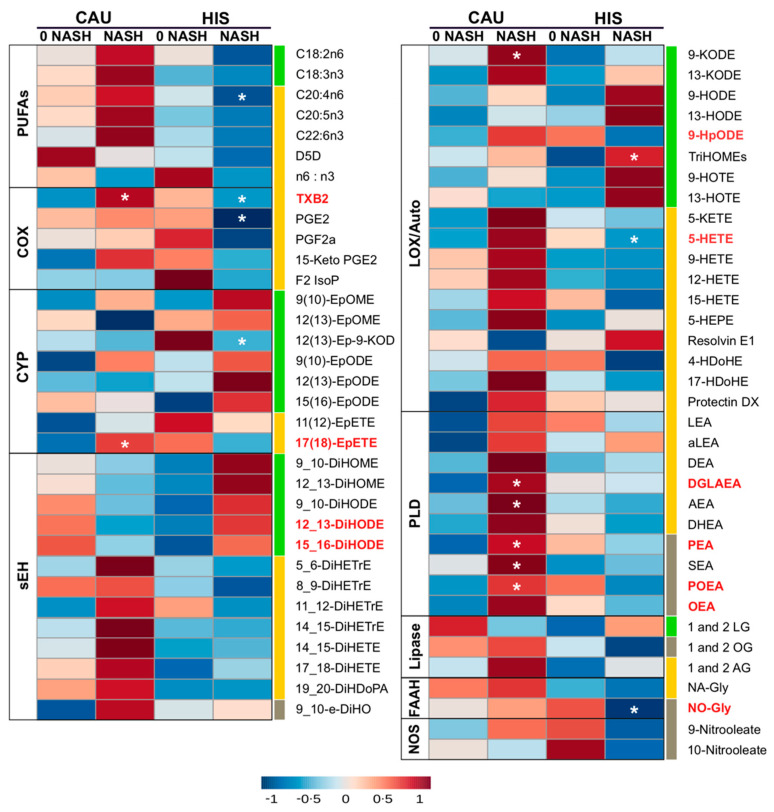
Heatmap illustrating fold changes and differences in plasma polyunsaturated fatty acids (PUFAs) and lipid mediators between non-alcoholic steatohepatitis (NASH) compared to NASH-free (0-NASH) in primary cohort. Fold changes are indicated by color and intensity, with red indicating an increase, and blue indicating a decrease. Lipids different in NASH vs. 0-NASH (*t*-test raw *p* < 0.05) are noted by (*). Ethnicity-related differences, or interaction (ethnicity × NASH) (ANCOVA raw *p* < 0.05) are marked with bold red color. C18 PUFAs and related lipids are marked with “green”; long chain-PUFAs and related lipids are marked with “yellow”; saturated fatty acids (SFA) and monounsaturated fatty acid (MUFA) related lipids are marked with “grey”. Means and *p*-values are detailed in Table S3. Fatty acids are described by number of carbons and double bounds of the fatty acyl moiety (i.e., C18:2n6). NASH (*n* = 6 Hispanic (HIS) and 3 Caucasian (CAU); 0-NASH (*n* = 4 HIS and 5 CAU). ADH, alcohol dehydrogenase; AEA, arachidonoyl ethanolamine; AG, arachidonoyl glycerol; DEA, docosatetraenyl ethanolamide; DGLEA, dihomo-gamma-linolenoyl ethanolamide; DH, dehydrogenase; DHEA, docosahexaenoyl ethanolamide; DiHDoPA, dihydroxydocosapentaenoic acid; DiHETE, dihydroxyeicosatetraenoic acid; DiHETrE, dihydroxyeicosatrienoic acid; DiHO, dihydroxyoctadecanoic acid; DiHODE, dihydroxyoctadecadienoic acid; DiHOME, dihydroxyoctadecenoic acid; Elov, fatty acid elongase; Ep-KODE, epoxyoxooctadecenoic acid; EpETE, epoxyeicosatetraenoic acid; EpODE, epoxyoctadecadienoic acid; EpOME, epoxyoctadecenoic acid; F2-IsoP, F2 isoprostanes; FAAH, fatty acid amide hydrolase; Fads, fattyaciddesaturase; HDoHE, hydroxydocosahexaenoic acid; HEPE, hydroxyeicosapentaenoic acid; HETE, hydroxyeicosatetraenoic acid; HODE, hydroxyoctadecadienoic acid; HOTE, hydroxyoctadecatrienoic acid; HpODE, hydroperoxyoctadecadienoic acid; KETE, keto-eicosatetraenoic; KODE, keto-octadecadienoic acid; LEA, Linoleyl ethanolamine; LG, linoleoylglycerol; LOX, lipoxygenase; NA-Gly, arachidonylglycine; NAPES, N-acylphosphatidyl ethanolamine-specific; NO-Gly; OEA, oleoyl ethanolamine; OG, oleoylglycerol; PEA, palmitoyl ethanolamine; PGE, prostaglandin E; PGF, prostaglandin F; POEA, palmitoleoyl ethanolamide; SEA, stearoyl ethanolamide; sEH, soluble epoxide hydrolase; TriHOME, trihydroxyoctadecaenoic acid; TXB, thromboxane.

We examined differences in plasma fatty acids and lipid mediators between 0-NASH and NASH (Figure 2 and Table S3). Compared to corresponding 0-NASH, 7 (11% of total detected) and 6 (9%) lipids were found different in NASH-HIS and NASH-CAU, respectively (raw *p*-value < 0.05). None passed the FDR-correction threshold. There were differentially altered lipids by ethnicity group (raw *p*-Interaction < 0.05), including 11 lipids (17%) and two enzymatic ratios. Three of these lipids and the two enzymatic ratios passed the FDR-correction threshold.

With the progression from 0-NASH to NASH, less marked differences in plasma PUFA profile were observed. Compared to 0-NASH, NASH-HIS showed a trend for lower plasma PUFAs, only affecting AA (raw *p*-value < 0.05). There was a trend for higher C18-PUFA derived alcohols, triol, epoxides and vicinal diols, with TriHOMEs being differentially higher (raw *p*-Interaction < 0.05). We also observed a trend for lower LC-PUFA derived lipid mediators, mainly affecting AA-alcohols, 5-hydroxyeicosatetraenoic acid (-HETE); thromboxane (TXB2); and prostaglandin (PGE2), with 5-HETE and TXB2 found differentially changed between ethnicities (raw *p*-Interaction < 0.05). Also, the oleic acid (OA)-derived N-oleoyl glycine levels were lower with NASH (raw *p*-Interaction < 0.05).

In NASH-CAU, there was a trend of higher C18, LC-PUFAs and downstream lipid mediators. Interaction (ethnicity × NASH) was shown with higher 9-HpODE, TXB2, and in EPA-epoxide, 17(18)-epoxyeicosatetraenoic acid (-EpETE) (raw *p*-Interaction < 0.05). There was an opposite trend for C18-PUFA derived vicinal diols that were higher in HIS and lower in CAU, compared to corresponding 0-NASH, with interaction (ethnicity × NASH) shown for 12,13- and 15,16-dihydroxyoctadecadienoic acid (-DiHODE) (raw *p*-Interaction < 0.05). Multiple sEH enzymatic indices were higher in HIS and lower in CAU, including 9_10-dihydroxyoctadecenoic acid (-DiHOME)/9(10)- epoxyoctadecenoic acid (-EpOME) and 9_10-DiHODE)/9(10)-epoxyoctadecadienoic acid (-EpODE) (raw *p*-Interaction < 0.05). Also, with NASH, there were higher levels of many eCBs, including dihomo-γ-linolenoylethanolamide, palmitoleoylethanolamide, palmitoleoylethanolamide, oleoyl-ethanolamide and N-oleoylglycine (raw *p*-Interaction < 0.05). Although PUFA changes are less marked in NASH, trends are consistent with changes seen in NAFL and support divergence in LC-PUFA profiles. It also highlights ethnicity-related differences in OXLs and eCBs associated with NASH progression. Given that NASH groups in both ethnicities had comparable NAS scores, this suggests the ethnicity-related differences observed with NASH are not likely driven by histological severity.

### 2.5. Plasma OXLs Profile Discriminates between Ethnicities with NASH

**Figure 3:**
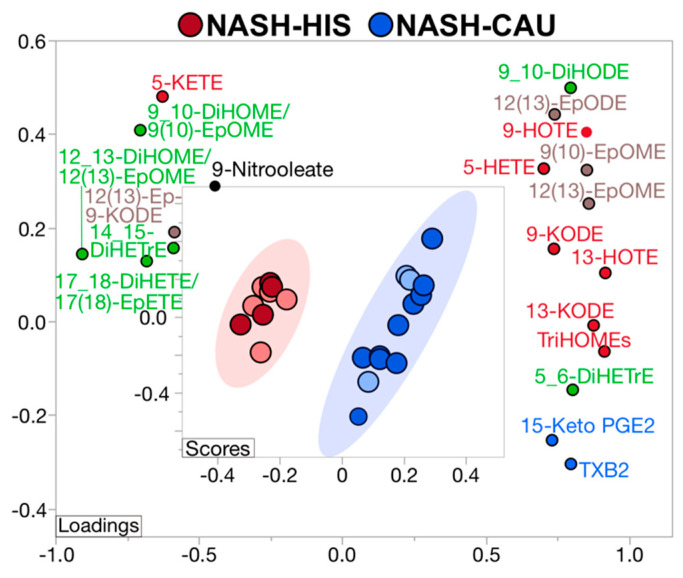
Supervised multivariate clustering model demonstrating ethnicity specific oxylipins profile discriminates Hispanic (HIS) and Caucasian (CAU) with non-alcoholic steatohepatitis (NASH) without advanced fibrosis. Score plot for NASH-HIS (red) vs. NASH-CAU (blue) when excluding stage 3 and 4 fibrosis. Light color represents subjects from primary cohort and dark color represents secondary cohort. The model was performed on all profiled lipids. Lipid mediators are colored according to metabolizing enzyme pathways, lipoxygenase, and autoxidation (red); cytochrome p450 epoxygenase (brown); and soluble epoxide hydrolase (green); cyclooxygenase (blue); other (black). The model was validated with leave-one-out cross validation. The Q^2^ and R^2^ are 0.99 and 0.98, respectively. Details on variable importance into the projection scores are shown in Table S4. NASH (*n* = 9 HIS and 13 CAU). DiHETE, dihydroxyeicosatetraenoic acid; DiHETrE, dihydroxyeicosatrienoic acid; DiHODE, dihydroxyoctadecadienoic acid; DiHOME, dihydroxyoctadecenoic acid; Ep-KODE, epoxyoxooctadecenoic acid; EpETE, epoxyeicosatetraenoic acid; EpODE, epoxyoctadecadienoic acid; EpOME, epoxyoctadecenoic acid; HETE, hydroxyeicosatetraenoic acid; HOTE, hydroxyoctadecatrienoic acid; KETE, keto-eicosatetraenoic; KODE, keto-octadecadienoic acid; PGE, prostaglandin E; TriHOME, trihydroxyoctadecaenoic acid; TXB, thromboxane.

A supervised PLS-DA was performed including all profiled lipids to examine if plasma OXLs profile can discriminate between ethnicities with NASH. The model demonstrated separation between HIS and CAU with 22 (49%) contributing lipids having VIP > 1. This indicates differences in OXLs profile characterizes HIS and CAU with NASH (Figure S3 and Table S4). The Q^2^ and R^2^ for the model were 0.62 and 0.72, respectively, indicating a fair reliability of the model. Of note, an overlap between ethnic groups was observed and subjects within this area shared advanced fibrosis (grade 3 and 4), indicating that HIS and CAU subjects with NASH and advanced fibrosis share similar plasma OXLs profile. Also, it suggests that advanced fibrosis may be attenuating the multivariate model. Therefore, we repeated the analysis after excluding subjects with advance fibrosis (Figure 3 and Table S4). As a result, the model exhibited complete separation between ethnicities in NASH with 20 (44%) lipids contributing to this difference (VIP > 1.0). The Q^2^, R^2^ were 0.99 and 0.98, respectively, indicating optimal prediction and reliability of the multivariate model.

In subjects with NASH and mild to moderate fibrosis, OXLs profiles showed opposite direction of change between ethnicities in some AA-derived mediators, with TXB2, 15-keto PGE2, 5-HETE and 5,6 DiHETrE being lower in HIS compared to CAU. Many OXLs derived via LOX pathway and/or auto-oxidative routes were lower in NASH-HIS, including TriHOMEs, 9- and 13-keto-octadecadienoic acid (-KODE), 9- and 13- hydroxyoctadecatrienoic acid (-HOTE), and 5-HETE. Some CYP-derived OXLs were lower in NASH-HIS, as 9(10)-EpOME, 12(13) EpOME and 12(13)-EpODE. Multiple sEH enzymatic indices were found higher in NASH-HIS, compared to NASH-CAU, including 12,13-DiHOME/12(13)-EpOME; 9,10-DiHOME/9(10)-EpOME; 17_18-DiHETE/17(18)-EpETE along with higher levels of the vicinal diol, 14,15-DiHETrE. These findings further confirm lower AA-derived OXLs in HIS with NASH, which are also characterized by lower LOX and higher sEH-derived lipid mediators compared to CAU. They also indicate that plasma OXL profiles can discriminate between ethnicities in NASH.

## 3. Discussion

This study is the first to examine targeted plasma PUFA, OXL, and eCB profiles with regards to ethnicity in a group of HIS and CAU subjects with obesity and biopsy-diagnosed NAFL and NASH. Our findings indicate that: (1) NAFL and NASH are characterized by ethnicity-related differences in plasma PUFA profiles, independent of histological severity; (2) Ethnicity-related differences in plasma OXLs profiles characterize NASH, independent of histological scores; (3) Plasma PUFA profile is altered in apparently healthy HIS, independent of obesity.

The hepatic and serum/plasma PUFA profiles are dysregulated in NAFLD [16,17,18,19,20]. Our results expand on these findings and show ethnicity-related differences in plasma PUFA profile in NAFL and NASH. With NAFL, both ethnicities showed higher ALA and LA levels, which were pronounced in CAU but not in HIS. This can be attributed to differences in the levels of these PUFAs in lean and healthy subjects, as HIS showed higher levels compared to their CAU counterpart. There was also a divergence in LC-PUFA profiles between ethnicities. CAU showed higher levels mainly affecting AA, while HIS displayed lower levels mainly affecting DHA and EPA. Consistent trends were shown with the progression from 0-NASH to NASH with arachidonic almost reaching significance (*p*-Interaction = 0.07). This is in line with our previous untargeted profiling in NAFL done on the same subjects showing higher C18-PUFAs in CAU, and a trend for lower LC-PUFA with tendencies shown for DHA and EPA in HIS [27]. The lack of significant difference in LC-PUFA in our previous analysis may be due to the semi-quantitative nature and the clustering statistical approach. Also in agreement with our current finding is the lower serum/plasma DHA and EPA levels reported in obese HIS, compared to non-HIS [28,29]. Together, this implies diminished plasma LC-PUFA characterizes obese HIS with NAFL and NASH. As diet affects circulating and tissue PUFA levels [30], the ethnicity-dependent differences in dietary intake of n-3 PUFAs which is reportedly lower in HIS could be responsible for these observed changes [29,31]. While we did not account for diet, our findings suggest a possible etiological role for it as we observed higher linoleic and ALA levels independent of obesity in lean healthy HIS compared to CAU. Beside diet, genetic variants in cluster region of fatty acid desaturases (FADS) can predict LC-PUFA serum/blood levels [32,33]. Single nucleotide polymorphisms (SNP) in FADS1 and FADS2, which encode fatty acid desaturases, were robustly associated with NAFLD [34,35]. Lower Δ−5 desaturase levels are reported in both NAFL and NASH [16,17,20]. Notably, SNPs in FADS that are associated with insufficient LC-PUFA biosynthesis present with high frequency in Amerindians, a subgroup of HIS [36]. However, genotype was not examined in current study, and both ethnicities had lower estimated Δ−5 desaturase activity with NAFL. Therefore, diet and/or genetic factors may contribute to the observed ethnicity-related PUFA alterations but need further assessment.

Other key findings include the ethnicity-related differences in OXLs and eCBs profiles. The COX pathway exerts pro-inflammatory effects as it catalyzes the conversion of AA to prostaglandin PGE2, thromboxane TXB2, and other fatty acid alcohols [10]. In animal models of NASH, the expression and activity of COX-2 were upregulated, and its inhibition ameliorated NAFL and NASH [37,38]. Previously, high TXB2 and PGE2 levels were reported in subjects with NAFL and NASH [22]. Findings in NASH-CAU from our secondary analysis are consistent with this literature, as TXB2 and 15-Keto PGE2 discriminated between ethnicities with NASH with higher levels in CAU and lower levels in HIS. When comparing within ethnicities, the progression from 0-NASH to NASH in HIS was marked with a trend for lower AA, almost reaching statistical significance (*p*-Interaction = 0.07) and downstream OXLs with TXB2 being differentially lower (*p*-Interaction < 0.05). These findings suggest ethnicity-related alterations in AA metabolism and downstream COX-derived OXLs in NASH.

Animal studies indicate a role for LOX pathways in NAFL and inflammation [39,40]. LOX pathways lead to the synthesis of fatty acid alcohols, ketones, hydroperoxides, and the specialized pro-resolving mediators (SPMs). With possible exceptions, n-6 PUFA derived alcohols are pro-inflammatory [10]. Under oxidative stress, PUFAs can also undergo auto-oxidation to form alcohols, ketones, hydroperoxides [11]. Previous studies reported higher LOX and auto-oxidation metabolites in NAFL and increased AA metabolites via LOX with the progression to NASH [18,22,23,41]. In our results, compared to control groups, NAFL and NASH in both ethnicities presented higher alcohols and ketones derived from C18-PUFAs, indicating an upregulated LOX pathway(s). In NAFL, we observed a positive correlation between some fatty acid alcohols and the oxidative stress markers, F2-isoprostanes and 9-HETE (Table S5), implying a contribution of non-enzymatic auto-oxidation. Interestingly, our secondary analysis showed many LOX derived OXLs being higher in NASH-CAU compared to NASH-HIS, with a similar trend found for the oxidative stress marker, 9-HETE (VIP = 0.98). Together, while LOX and oxidative pathways are upregulated with NAFL in both ethnicities, the magnitude of these alterations is lesser in HIS with NASH, compared to CAU. Based on this finding, we reasoned that LOX, and possibly oxidative stress, may be pivotal for NASH severity in CAU, and to a lesser extent in HIS.

CYP enzymes catalyze the synthesis of fatty acids epoxides and alcohols. In general, fatty acid alcohols are pro-inflammatory, and epoxides are anti-inflammatory and transient, and are hydrolyzed by the action of sEH to form inactive or less active vicinal diols [10,42]. A role for sEH in NAFLD progression is indicated by animal studies, showing that sEH inhibition improves NAFL, NASH, and fibrosis [42]. In subjects with NASH, compared to NAFL, AA derived vicinal diols are higher [22]. Our results show, with the progression to NASH, an ethnicity-dependent opposite trend for vicinal diols derived from C18-PUFA, which were higher in HIS and lower in CAU. Some of these vicinal diols and sEH enzymatic indices showed interaction (ethnicity × NASH) and were found higher in NAHS-HIS and lower in NASH-CAU, compared corresponding 0-NASH. This may suggest higher activity of sEH in NASH-HIS. Our secondary analysis also shows higher ratios of multiple sEH enzymatic indices in HIS compared to CAU, and lower C18-PUFA epoxides possibly due higher hydrolysis rate. NASH-CAU showed higher levels of many PUFA epoxides, compared to NASH-HIS, indicating upregulated CYP pathway(s) and/or less hydrolysis. Together, our finding highlights ethnicity-related differences in sEH activity that was higher in HIS with NASH.

Extensive evidence from animal studies indicates a role for eCB system in NAFL, mitochondrial dysfunction and inflammation and fibrosis [43,44]. In NAFL, both ethnicities had higher levels of several eCBs. However, with the progression to NASH, many eCBs were higher in CAU and lower in HIS as compared to corresponding 0-NASH (raw *p*-Interaction < 0.05). We also observed levels of the OA-derived mediators N-oleoyl glycine, and oleoylethanolamide. These observations could not be examined in our secondary analysis as we detected limited numbers of eCBs and did not profile for fatty acids. Nevertheless, this may indicate ethnicity-related variations in eCBs profiles and OA metabolism with NAFLD in HIS that need to be further examined.

Our findings corroborate the epidemiological evidence indicating ethnicity as one variable affecting the association between PUFAs and cardiometabolic risks [29,45,46]. In fact, the observed ethnicity-related alterations may be relevant to NAFLD severity. EPA and DHA modulate hepatic fatty acid oxidation, de novo lipogenesis, redox balance and inflammation via direct interaction with nuclear receptors and transcription factors [47]. These LC-PUFAs are also precursors to potent SPMs which drive inflammatory resolution [10]. Also, the pro-inflammatory cascade of AA via COX is necessary for the biosynthesis of SPMs and initiating inflammatory resolution [13,48]. Therefore, a diminished level of these PUFAs may abolish anti-steatogenic and anti-inflammatory mechanisms. Likewise, a higher sEH activity may result in deactivation of anti-inflammatory PUFA epoxides [10,42]. Interestingly, our findings suggest that upregulated LOX pathway(s) may be imperative to NASH severity in CAU with a lesser extent in HIS. Collectively, we postulate that the observed ethnicity-related changes translate to the more advanced NASH histological presentation seen in HIS. Of note, these changes are independent of fibrosis or NAS scores, in fact, histology adjustment resulted in stronger differences in both analyses, implying that subjects with advanced fibrosis may share similar lipidomic profile.

Our findings have clinical/diagnostic implications. Given liver biopsy risks and limitations [49], there is an ongoing search for noninvasive biomarker for NAFLD, with multiple biomarkers have been recently proposed including betatrophin and fetuin-A [50,51]. Also, several AA- derived OXLs were shown to predict NASH including higher levels of 5- and 15-HETE, PGE2, and some vicinal diols [18,22]. While our findings in NASH-CAU show trends consistent with current literature, findings in HIS indicate otherwise. Ethnicity-related differences in plasma metabolomic profile have been reported before in diabetes, Alzheimer’s disease, and bladder cancer [52,53,54]. We propose that ethnicity-specific plasma signature may characterize NASH. In fact, utilizing ethnicity-related variations in plasma lipidomic profile may be instrumental for the enhanced precision of such diagnostic tools. If further verified, it will serve as a much-needed non-invasive tool aiding in clinical practice for early detection of NASH in both HIS and CAU populations. It can also pave the road for examination of ethnicity-specific lipidomic signatures in other ethnicities as the Asian and African American populations. On another note, a role of EPA and DHA supplementations in improving NAFLD and its risk factors is supported by clinical evidence [55,56]. Also, growing data indicate the utility of sEH inhibitors in NASH treatment [42,57,58]. Therefore, evaluating these interventions for NASH treatment seems warranted, particularly in the HIS population.

This “proof-of-concept” analysis is based on a small, single-center study. The limited sample size may have compromised the correction for multiple testing in the primary analysis. However, findings from the secondary analysis were consistent and the multivariate model is validated for overfitting and predictability. Other strengths include biopsy-characterized NAFL and NASH and analysis adjusted for BMI and histology. While NAFLD prevalence is reported to be higher in males compared to females [59,60,61], we could not examine sex differences due to small sample size. However, we did adjust for sex as a covariate.

In conclusion, we performed targeted lipidomic profiling for PUFAs and related lipid mediators with regards to ethnicity. Results show ethnicity-related divergence in LC-PUFA and downstream OXLs profiles with NAFL and NASH progression, independent of histological scores. Our secondary analysis indicates that in NASH and compared to CAU, HIS are characterized by lower levels of AA derived OXLs, lower LOX with an upregulated sEH pathway(s). These lipidomic differences may be relevant to the ethnicity-related disparity reported in NAFLD rate and severity and are worth further investigations. Our findings suggest ethnicity-specific lipidomic signature may characterize NASH. Although preliminary, these novel observations support the need for larger validation studies.

## 4. Subjects and Methods

### 4.1. Subjects and Samples

In this retrospective/ prospective cohort study (Figure S4), all subjects self-reported ethnicity as either HIS or CAU. HC subjects (*n* = 22) were recruited via public posts. Plasma and liver samples form bariatric surgery patients with medically complicated obesity were retrieved from the biobank repository of the Division of Gastroenterology and Hepatology, UC Davis Medical Center. The primary cohort (*n* = 18) consisted of subjects with NAFL and various degrees of necroinflammation. Only subjects with NASH were included in the secondary analysis (*n* = 9) and this cohort was expanded with prospectively collected subjects diagnosed with NASH (*n* = 20). Subject inclusion and exclusion criteria and details on data collection are described elsewhere [25]. Briefly, plasma samples were collected preoperatively after an overnight fast, and liver tissue samples were collected by biopsy performed during bariatric surgery. Liver histopathological evaluations were performed in a blinded fashion in the UC Davis Medical Center Department of Pathology, and samples were scored according to the NASH Clinical Research Network (NASH-CRN) histology system, The NAFLD Activity Score (NAS) and fibrosis scores were calculated [26]. NASH diagnosis was determined using a diagnostic algorithm based on steatosis, inflammation, and fibrosis scores [27]. All subjects were consented and the Institutional Review Board at the University of California, Davis approved the study protocol (# 856052).

### 4.2. Plasma Targeted Lipidomic Analysis

Quantitative lipidomic profiling of PUFAs, OXLs and NAEs was performed by ultra-high-performance liquid chromatography-electrospray ionization-tandem mass spectrometry (UPLC-ESI-MS/MS) (S2), as previously described [62]. Briefly, plasma samples were enriched with deuterated surrogates, isolated by liquid/liquid extraction, and separated and quantified by UPLC-ESI-MS/MS. In the primary analysis, ESI-polarity switching facilitated the simultaneous detection of eCBs (positive mode) and oxylipins and PUFAs (negative mode) on an API 6500 QTRAP (AB Sciex, Framingham, MA, USA). Metabolites were quantified against authentic analytical standards with 6-to-10-point calibration curves and calculated concentrations were corrected for analytical surrogate recovery. This method detected 5 PUFAs and 66 lipid mediators, including 10 eCBs and 46 OXLs.

The secondary analysis was performed on an API 4000 QTrap (AB Sciex) and restricted to the negative mode electrospray ionization to increase the power of the OXLs discovery. This approach detected 46 OXLs and two nitrolipids. Details on the analysis protocols and reported data are available on the Metabolomics Workbench (http://www.metabolomicsworkbench.org, accessed on 13 June 2021). ID numbers (ST000977 and ST001845). Analyses were carried out at the UC Davis West Coast Metabolomics Center. In this manuscript, abbreviations used for OXLs and eCBs follow standard consensus and are detailed with lipid identifiers in Table S6.

### 4.3. Statistical Analysis

Statistical analyses were performed using JMP Pro 14.1 (SAS Institute Inc., Cary, NC; http://www.jmp.com, accessed on 10 March 2021). Outliers were identified and excluded using “robust Huber M test”. Lipids with >30% missing data were excluded. Missing data were imputed by “multivariate normal imputation”. Data normality was achieved by Johnson’s transformation. After data processing, and to determine if subjects with stage 4 fibrosis (*n* = 2) are biological outliers, we employed principal component analysis (PCA). As a result, no outliers were detected (Figure S5).

Non-normalized data were used to calculate metabolite geometric means. Fold change (FC) was calculated for each ethnicity separately as (A − B)/B where A is the mean of (NAFL or NASH) and B is the mean of (HC or 0-NASH). A FC > 0 indicates an increase and <0 indicates decrease and ± 20% FC was set as a threshold. Student’s *t*-test of Johnson normalized data was used to examine differences between (NAFL vs. HC) and (0-NASH vs. NASH) in each ethnicity. Full factorial analysis of covariates (ANCOVA) was employed to evaluate the interaction of ethnicity × health status. This model included ethnicity (HIS or CAU), health status (NAFL or HC; 0-NASH or NASH), ethnicity × health status interaction as fixed effects, with age and sex as covariates. To check any effect of fibrosis or advance NAS score on the differences observed, we repeated the analysis on a subset of histology-matched subjects (*n* = 5 HIS and *n* = 5 CAU). Pathways/network visualization with fold change and *p*-values were plotted using Cytoscape 3.8.2 (https://cytoscape.org, accessed on 22 May 2021) [63]. Mean differences were considered likely at *p* < 0.05. To adjust for false discovery rate (FDR), Benjamini-Hochberg FDR correction was performed [64]. A *q* = 0.2 was set as a threshold, given the pilot nature and small sample size of the study.

For the secondary analysis, raw data were auto-scaled to correct for batch effect (Figure S6) [65]. Lipids affected by batch were excluded (three lipids) and data were normalized by Johnson’s transformation. Partial least square-discriminant analysis (PLS-DA) was performed to discriminate ethnicities in NASH subjects with leave- one-out cross validation (LOOCV) [66]. An R^2^ and Q^2^ > 0.5 are acceptable values to indicate reliability of the model in explaining differences between groups [67]. A variable importance in projection (VIP) score of >1.0 was set as a threshold for variable selection. To check any effect of advanced fibrosis, we repeated the analysis after excluding subjects with fibrosis grade 3 and 4 (*n* = 3 HIS and *n* = 4 CAU).

## Figures and Tables

**Table 1:** Demographic, clinical, and histological characteristics of study subjects in primary analysis.

	NAFL-HIS	NAFL-CAU	*p*-Value *
Plasma, *n* (F/M)	10 (7/3)	8 (4/4)	-
Age (years)	47 ± 15	50 ± 18	0.6
DM, yes (%)	4 (40)	4 (50)	1
FBG mmol/L	101 ± 14	94 ± 13	0.3
Cholesterol (mg/dL)	172 ± 28	166 ± 34	0.7
TG (mg/dL)	135 ± 79	129 ± 70	0.8
HDL (mg/dL)	44 ± 6	43 ± 8	0.7
LDL (mg/dL)	107 ± 24	99 ± 35	0.4
HbA1c (%)	6 ± 1	6 ± 1	0.7
AST (U/L)	31 ± 13	26 ± 9	0.6
ALT (U/L)	40 ± 27	31 ± 11	0.9
Platelet	280 ± 84	302 ± 110	0.8
NAS	3 ± 3	3 ± 1	0.7
Steatosis (%)			
<5%	4 (40)	1 (12.5)	0.9
5 to ≤33%	3 (30)	5 (62.5)	
34 to ≤66 %	2 (20)	2 (25)	
>66%	1 (10.0)	0 (0)	
Inflammation (%)			
None	3 (30)	1 (12)	0.6
Mild	1 (10)	4 (50)	
Moderate	4 (40)	3 (38)	
Severe	2 (20)	0 (0)	
Ballooning (%)			
None	4 (40)	5 (62)	0.3
Few	5 (50)	3 (38)	
Many	1 (10)	0 (0)	
Fibrosis (%)			
None	7 (70)	7 (88)	0.3
1A	0 (0)	1 (12)	
2	1 (10)	0 (0)	
4	2 (20)	0 (0)	

General characteristics of NAFL group in both ethnicities shown as percent (for categorical data) and mean ± SEM (for nominal data). Comparisons were performed by *t*-test (nominal) or chi-square test (categorical). (*) NAFL-HIS vs. NAFL-CAU.

**Table 2:** Demographic, clinical, and histological characteristics of NASH subjects in secondary analysis.

	NASH-HIS	NASH-CAU	*p*-Value *
Plasma, *n* (F/M)	12 (9/3)	17 (11/6)	-
Age (years)	49 ± 8	50 ± 13	0.8
BMI (Kg/m^2^)	41 ± 8	37 ± 7	0.6
NAS	5 ± 2	5 ± 1	0.9
Steatosis (%)			
<5%	2 (17)	0 (0)	0.4
5 to ≤33%	4 (33)	7 (41)	
34 to ≤66 %	3 (25)	4 (24)	
>66%	3 (25)	6 (35)	
Inflammation (%)			
None	0 (0)	0 (0)	0.1
Mild	2 (17)	3 (18)	
Moderate	6 (50)	13 (76)	
Severe	4 (33)	1 (6)	
Ballooning (%)			
None	0 (0)	0 (0)	0.6
Few	8 (67)	13 (76)	
Many	4 (33)	4 (24)	
Fibrosis (%)			
None	5 (41)	4 (23)	0.8
1a, b, c	2 (17)	6 (35)	
2	2 (17)	3 (18)	
3	2 (17)	2 (12)	
4	1 (8)	2 (12)	

General characteristics of subjects included in the secondary analysis shown as percent (for categorical data) and mean ± SEM (for nominal data). Comparisons were performed by *t*-test (nominal) or chi-square test (categorical). (*) NASH-HIS vs. NASH-CAU.

## Data Availability

Analysis protocol and data are available at the Metabolomics Workbench (http://www.metabolomicsworkbench.org, accessed on 13 June 2021), study ID numbers (ST000977 and ST001845).

## Supplementary Materials

The following supporting information can be downloaded at: https://www.mdpi.com/article/10.3390/metabo12020192/s1, Table S1: Targeted quantification data table for comparisons of HIS and CAU obese subjects with NAFL vs. lean HC, Table S2: Demographic, clinical, and histological characteristics of histology-matched subjects, Table S3: Targeted quantification data table for comparisons of HIS and CAU with NASH vs. 0-NASH, Table S4: Targeted quantification data table for comparisons of HIS and CAU with NASH in the secondary analysis, Table S5: Pearson’s correlation analysis between LOX metabolites and markers of oxidative stress, Table S6: Details on detected lipid class and identifiers, Figure S1: Venn-Diagram illustrating overlapping and unshared differences in polyunsaturated fatty acids (PUFAs) and lipid mediators between non-alcoholic fatty liver (NAFL) compared to healthy control (HC) in both ethnicities, Figure S2: Differences in plasma PUFA and lipid mediators between Hispanic (HIS) and Caucasian (CAU) in lean healthy subjects, Figure S3: Supervised multivariate clustering model demonstrating ethnicity specific oxylipins profile discriminates Hispanic (HIS) and Caucasian (CAU) with non-alcoholic steatohepatitis (NASH) with advanced fibrosis, Figure S4: Flow chart illustrating subject recruitment details for primary and secondary analysis, Figure S5: Principal component analysis (PCA) illustrating outliers before (left) and after (right) data normalization, Figure S6: Principal component analysis (PCA) illustrating the unsupervised clustering of samples from primary (red) and secondary cohort (green) shown before (left) and after (right) batch correction.
