# Supplementary figures and images for: Plasma Oxylipin Profile Discriminates Ethnicities in Subjects with Non-Alcoholic Steatohepatitis: An Exploratory Analysis
Source: Metabolites. 2022 Feb 19;12(2):192. doi: 10.3390/metabo12020192 (PMC8875408; doi:10.3390/metabo12020192)

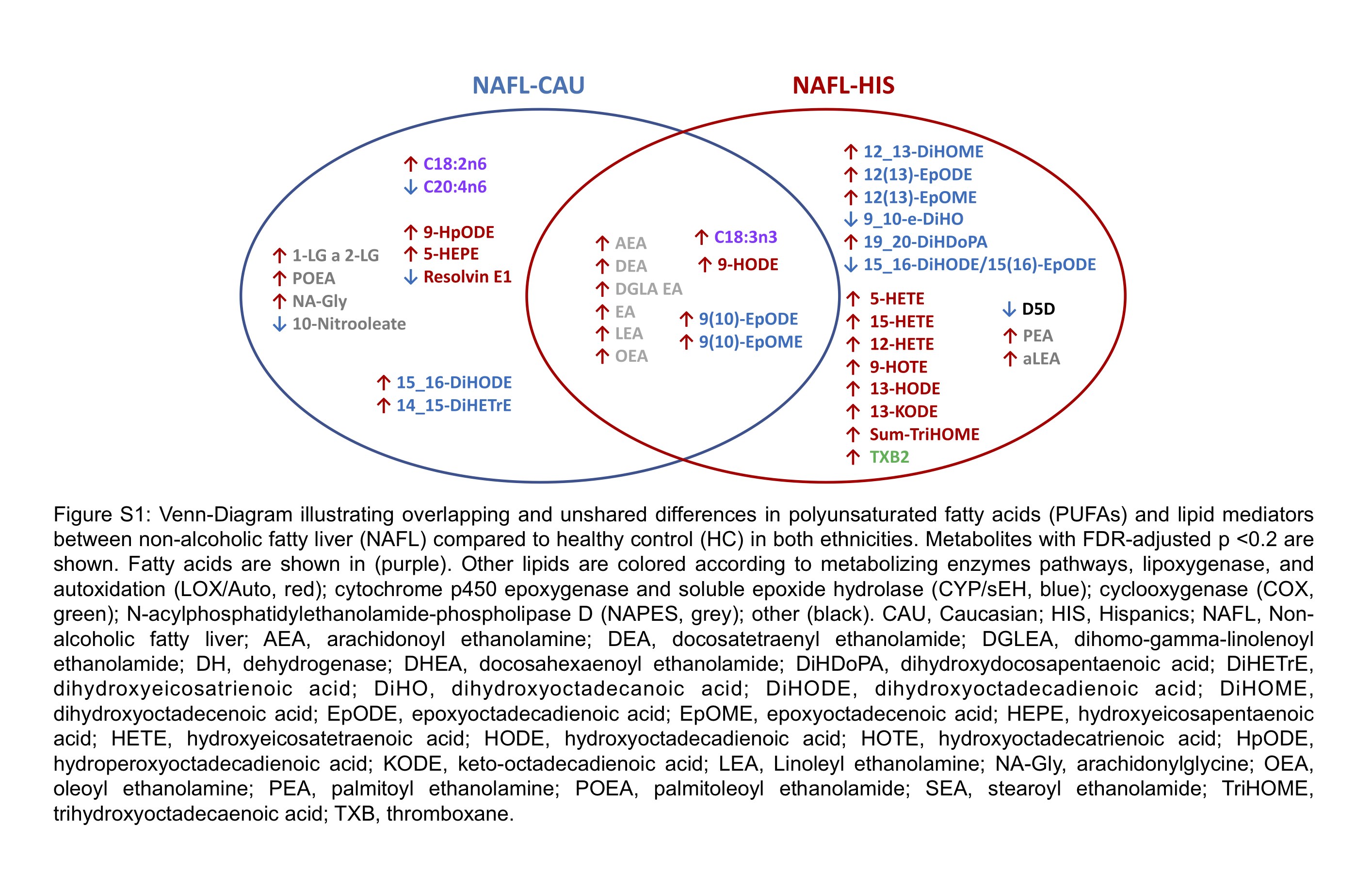

Supplement: Supplementary file 1 [file metabolites-12-00192-s001.zip › metabolites-1606194-supplementary/Fig_S1.png]

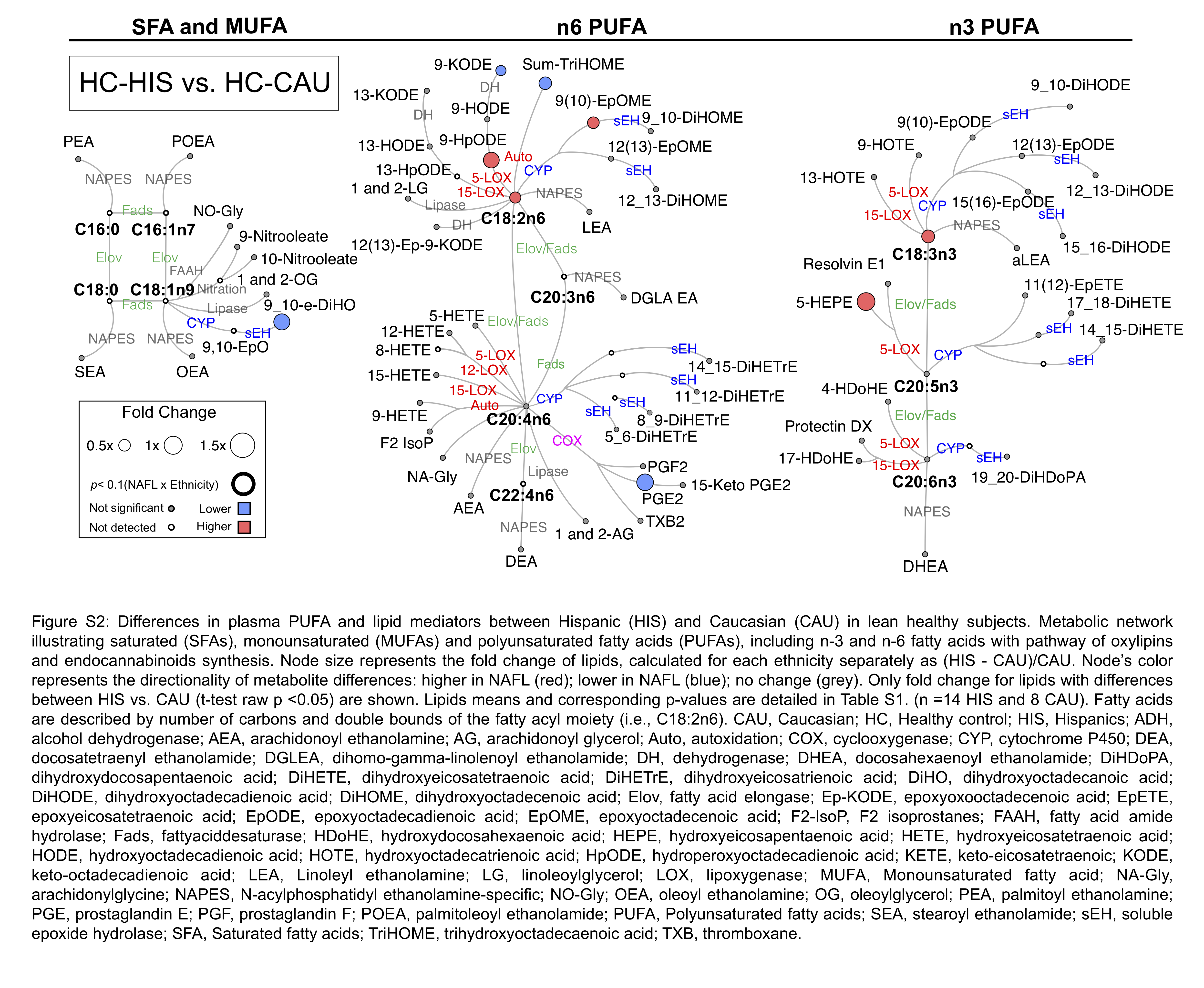

Supplement: Supplementary file 1 [file metabolites-12-00192-s001.zip › metabolites-1606194-supplementary/Fig_S2.png]

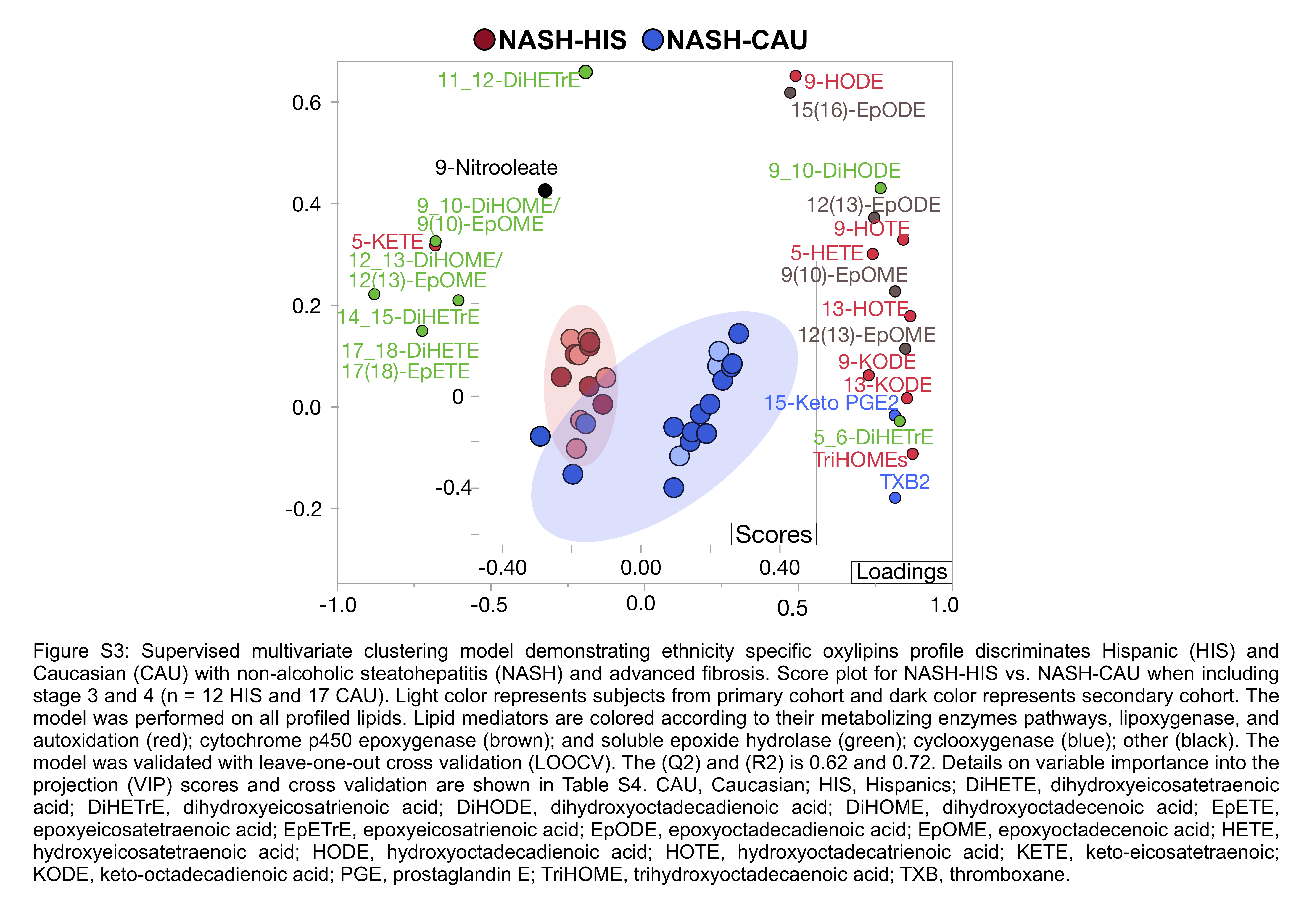

Supplement: Supplementary file 1 [file metabolites-12-00192-s001.zip › metabolites-1606194-supplementary/Fig_S3.png]

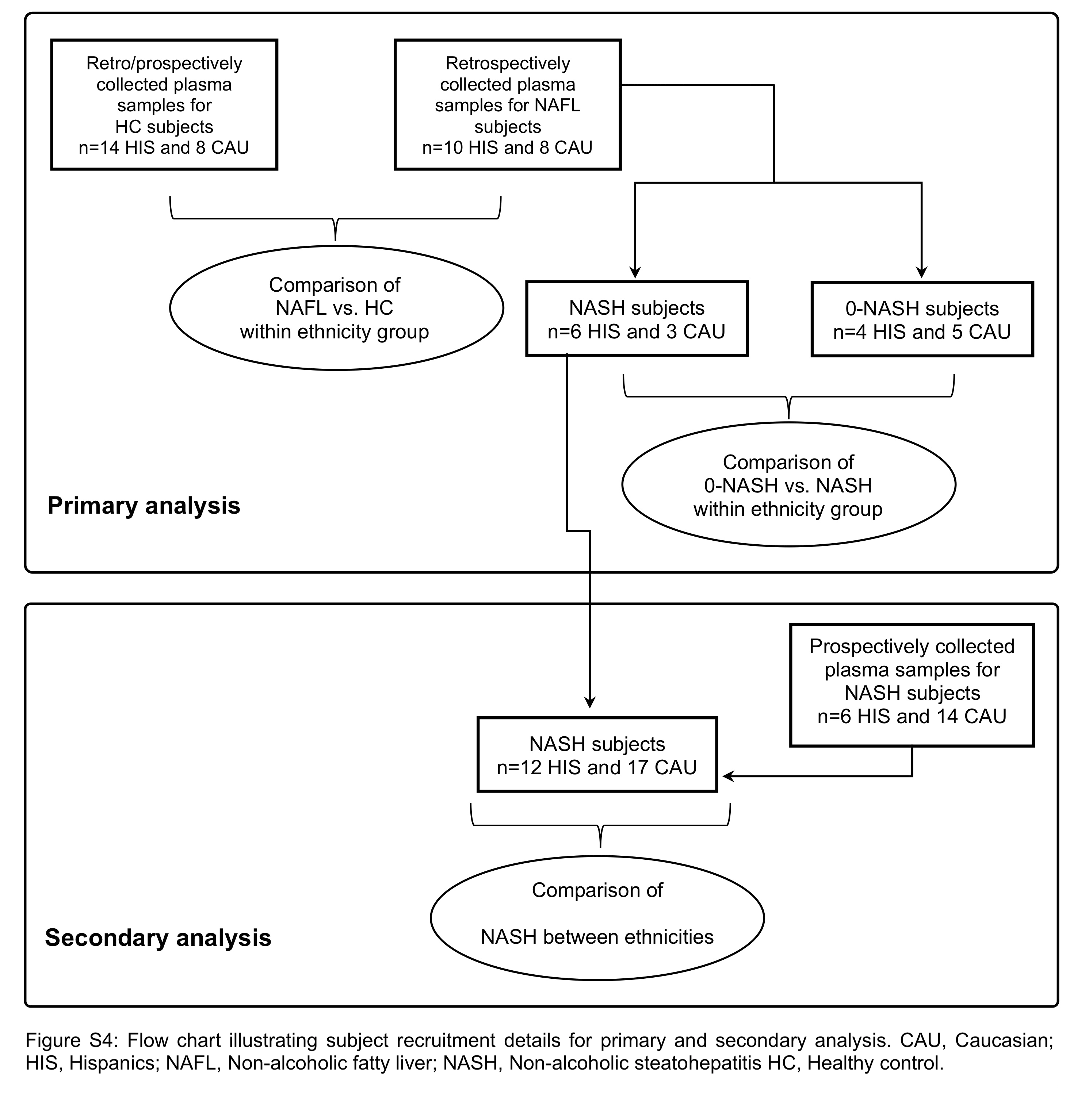

Supplement: Supplementary file 1 [file metabolites-12-00192-s001.zip › metabolites-1606194-supplementary/Fig_S4.png]

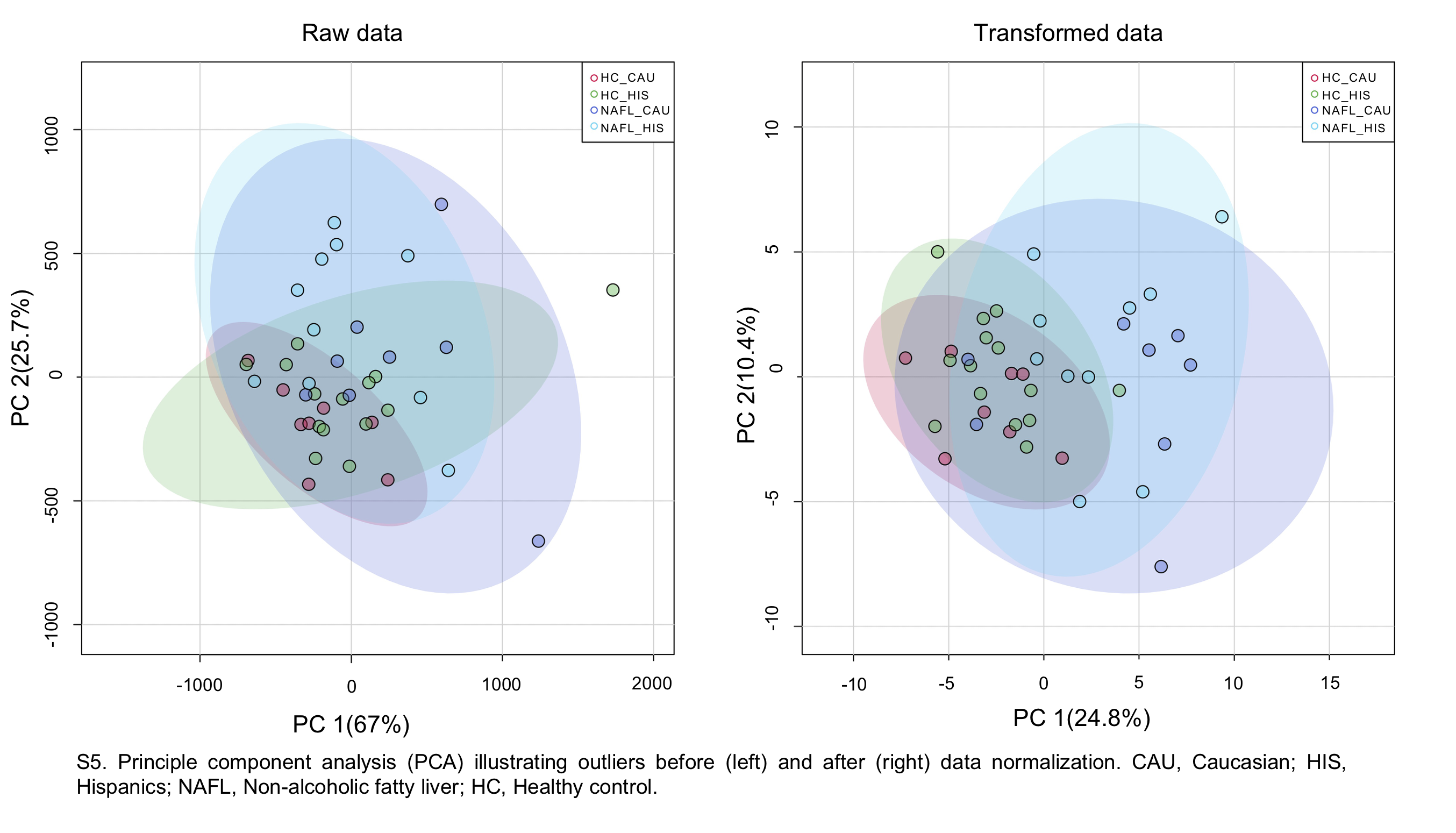

Supplement: Supplementary file 1 [file metabolites-12-00192-s001.zip › metabolites-1606194-supplementary/Fig_S5.png]

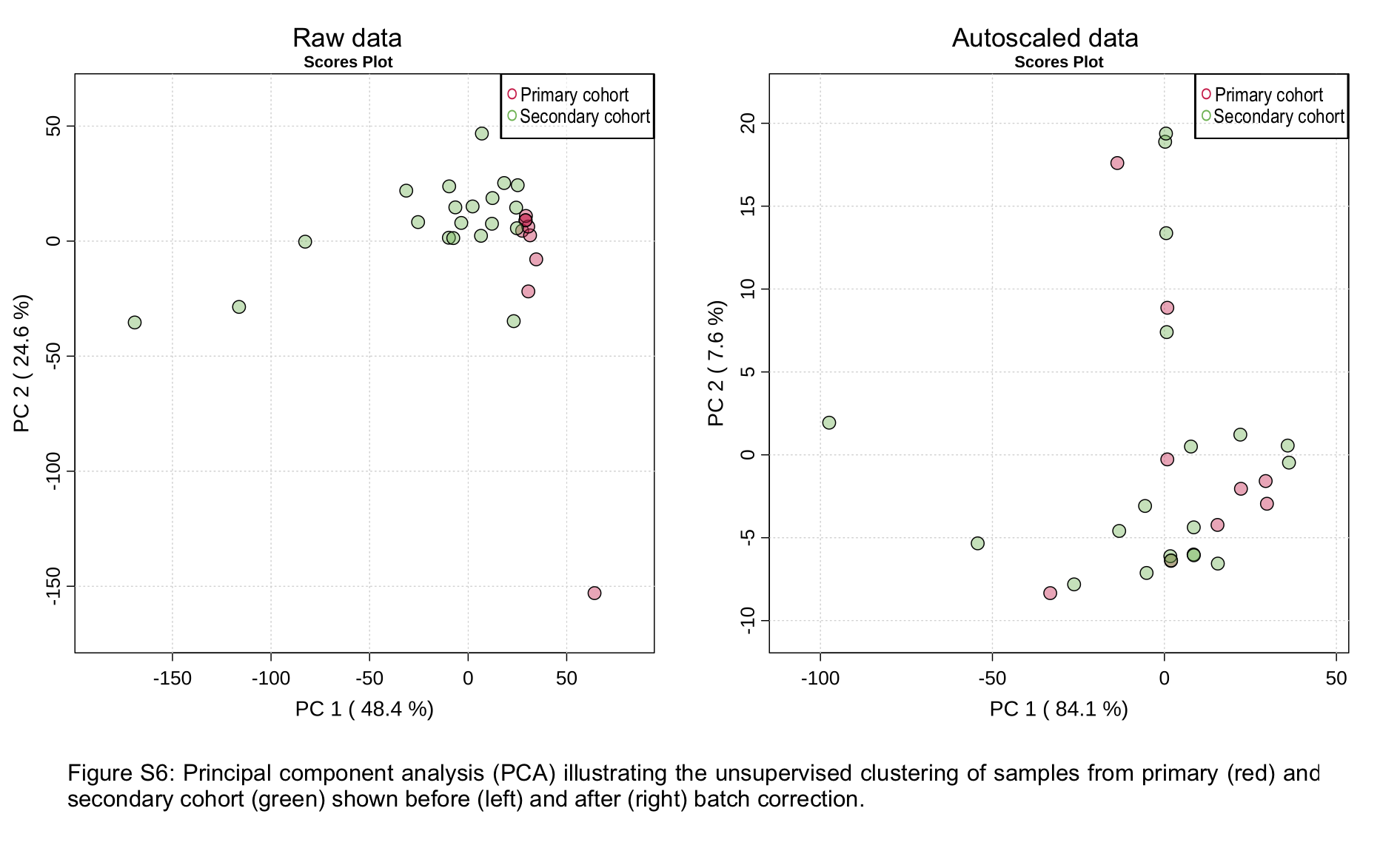

Supplement: Supplementary file 1 [file metabolites-12-00192-s001.zip › metabolites-1606194-supplementary/Fig_S6.png]
